# Embolization for acute nonvariceal bleeding of upper and lower gastrointestinal tract: a systematic review

**DOI:** 10.1186/s42155-023-00360-3

**Published:** 2023-03-29

**Authors:** Corrado Ini’, Giulio Distefano, Filippo Sanfilippo, Davide Giuseppe Castiglione, Daniele Falsaperla, Francesco Giurazza, Cristina Mosconi, Francesco Tiralongo, Pietro Valerio Foti, Stefano Palmucci, Massimo Venturini, Antonio Basile

**Affiliations:** 1 Department of Medical Surgical Sciences and Advanced Technologies “G.F. Ingrassia”, University of Catania —Radiology I Unit, University Hospital Policlinico “G. Rodolico-San Marco”, Via Santa Sofia 78, 95123 Catania, Italy; 2 Department of Anaesthesia and Intensive Care, A.O.U. ‘Policlinico-Vittorio Emanuele’, Catania, Italy; 3 Vascular and Interventional Radiology Department, Cardarelli Hospital, Via A. Cardarelli 9, 80131 Naples, Italy; 4 Department of Radiology, IRCCS Azienda Ospedaliero—Universitaria di Bologna, 40138 Bologna, Italy; 5 Diagnostic and Interventional Radiology Department, Circolo Hospital, Insubria University, Viale Luigi Borri 57, 21100 Varese, Italy

**Keywords:** Hemorrhage, Haemorrhage, Gastrointestinal, Embolization, Embolisation, Embolotherapy

## Abstract

**Background:**

Acute non-variceal gastrointestinal bleedings (GIBs) are pathological conditions associated with significant morbidity and mortality. Embolization without angiographic evidence of contrast media extravasation is proposed as an effective procedure in patients with clinical and/or laboratory signs of bleeding. The purpose of this systematic review is to define common clinical practice and clinical and technical outcomes of blind and preventive embolization for upper and lower gastrointestinal bleeding.

**Main body:**

Through the PubMed, Embase and Google Scholar database, an extensive search was performed in the fields of empiric and preventive embolization for the treatment of upper and lower gastrointestinal bleedings (UGIB and LGIB). Inclusion criteria were: articles in English for which it has been possible to access the entire content; adults patients treated with empiric or blind transcatheter arterial embolization (TAE) for UGIB and/or LGIB. Only studies that analysed clinical and technical success rate of blind and empiric TAE for UGIB and/or LGIB were considered for our research. Exclusion criteria were: recurrent articles from the same authors, articles written in other languages, those in which the entire content could not be accessed and that articles were not consistent to the purposes of our research. We collected pooled data on 1019 patients from 32 separate articles selected according to the inclusion and exclusion criteria. 22 studies focused on UGIB (total 773 patients), one articles focused on LGIB (total 6 patients) and 9 studies enrolled patients with both UGIB and LGIB (total 240 patients). Technical success rate varied from 62% to 100%, with a mean value of 97.7%; clinical success rate varied from 51% to 100% with a mean value of 80%. The total number of complications was 57 events out of 1019 procedures analysed.

**Conclusion:**

TAE is an effective procedure in the treatment of UGIB patients in which angiography does not demonstrate direct sign of ongoing bleeding. The attitude in the treatment of LGIBs must be more prudent in relation to poor vascular anastomoses and the high risk of intestinal ischemia. Blind and preventive procedures cumulatively present a relatively low risk of complications, compared to a relatively high technical and clinical success.

## Introduction

Acute non-variceal gastrointestinal bleedings (GIBs) are relatively frequent pathological conditions associated with significant morbidity and mortality, mainly in those patients without or with delayed treatment or with comorbidities. Conventionally, GIBs are divided into upper gastrointestinal bleeding (UGIB - the source of bleeding is proximal to the ligament of Treitz) and lower gastrointestinal bleeding (LGIB - the source of bleeding is located downstream of the duodenum-jejunal junction, including the small intestine, colon, rectus and anus). UGIBs are more frequent than LGIBs and represent 80% of acute GI bleeds, with an annual incidence of approximately 70 to 150 per 100,000 inhabitants, a higher prevalence in males and elderly subjects and with estimated mortality rates between 7% and 10%, and up to 35% in patients with comorbidities (Kumar and Mills 2011; Parente et al. 2005). Peptic ulcers are the most common cause of UGIB (30-50%); other causes of UGIB include erosive gastritis and/or duodenitis, oesophagitis (10%), varices (2-9 %), Mallory-Weiss tears (5%), malignancies (2-5%) and vascular malformations (5%). LGIB represents 20 to 25 % of all gastrointestinal bleedings. The main causes of LGIB are diverticular bleedings (30-50%), angiodysplasia (3-10%), ischemic colitis (2-9%), infectious colitis, chronic inflammatory bowel disease (6-30%), colon cancer, hemorrhoids and post-surgical bleedings (post-polypectomy, post-biopsy) (Ahmed and Stanley 2012; Lee and Laberge 2004). Clinical manifestations depend on the location of the bleeding, the extent, and the duration; comorbidities and, above all, cardiovascular stability play a fundamental role in the clinic and therapeutic management (Gerson et al. 2015). Chronic gastrointestinal bleeding up to 100ml / day can remain asymptomatic in the stable subject, over 500 ml / day leads to initial signs of hypovolemia (tachycardia and orthostatic hypotension), while the loss of 15% of the blood volume leads to the haemorrhagic shock (Ahmed and Stanley 2012). Stratification of clinical and rebleeding risk has been well described for non-variceal UGIBs. In 1975 Forrest et al. first described a correlation between the endoscopic finding of active bleeding and mortality (Forrest et al. 1974); subsequently, Rockal et al. developed a rather complex score in which clinical parameters (age, signs of shock, co-morbidities, endoscopic diagnosis) and bleeding characteristics were taken into account to calculate the risk of re-bleeding and mortality before and after endoscopy (Rockall et al. 1996); more recently, Blatchford et al. proposed a score based on the evaluation of haemoglobin, blood urea, pulse, and systolic blood pressure, as well as presentation with syncope or melaena, and evidence of hepatic disease or cardiac failure (Blatchford et al. 2000). AIMS65 is a new score introduced in 2011 to assess the risk of in-hospital mortality (Kim et al. 2019). The new AIMS65 seems to be comparable to the Rockal score and the Blatchford score in predicting mortality and the risk of re-bleeding in patients with UGIB, and some authors recommend its use in place of the previous ones for greater practicality in the calculation (Kim et al. 2019). The management of GI bleeding often involves several professional figures, including emergency medicine physicians, general surgeons, endoscopists, radiologists and in recent decades an increasing role has been recognized to interventional radiologists. In 2017 the American College of Radiology dictates the guidelines for management of UGIB highlighting the importance of performing visceral arteriography in the diagnostic and therapeutic management of UGIBs after an unsuccessful endoscopy or in case of contraindication to endoscopy (Expert Panels on Vascular Imaging and Gastrointestinal Imaging et al. 2017). LGIBs in most cases are self-limiting and usually have a less dramatic clinical presentation than UGIBs; in patients with LGIB, CT scan examination is the gold standard imaging technique to diagnose this condition; in many hospitals colonoscopy is the first therapeutic approach, however often fails due to poor patient preparation and copious bleeding (Laine 2020). Therefore, interventional radiology should be considered in patient with high-risk features and ongoing bleeding and in which colonoscopy is not available or failed (Darcy et al. 2014; Strate and Gralnek 2016). Since endoscopic treatment does not successfully control bleeding or rebleeding occurring in 8%–25% of the patients, many institutions have used TAE as the first line therapy for the management of upper gastrointestinal bleeding. However, gastrointestinal bleeding site is often difficult to detect by angiography because it is frequently intermittent due to unstable bleeding, hypotension, tamponade of the bleeding vessel by the hematoma formed and vasospastic changes of the vessels involved. Among therapeutic options for these patients, interventional radiologists could perform empiric TAE, defined as embolization without angiographic direct signs of bleeding, or preventive TAE, defined as embolization of pathological vessel after successful endoscopic treatment in patients with high risk of rebleeding. In the literature the following definitions need to be clarified, and they have been used as keywords for the bibliographic research for the drafting of this work. The term “overt GIB” refers to clinically evaluable bleeding for which the source of bleeding has been identified on imaging; "obscure GIB" means bleeding for which the origin has not been localized through second-level imaging methods; “occult GIB” means a condition known only through laboratory data (anemia, fecal occult blood) (Ahmed and Stanley 2012). The purpose of this paper is to review common clinical practice, the clinical and technical outcomes of blind and preventive embolization for UGIBs and LGIBs.

## Materials and methods

**Fig. 1:**
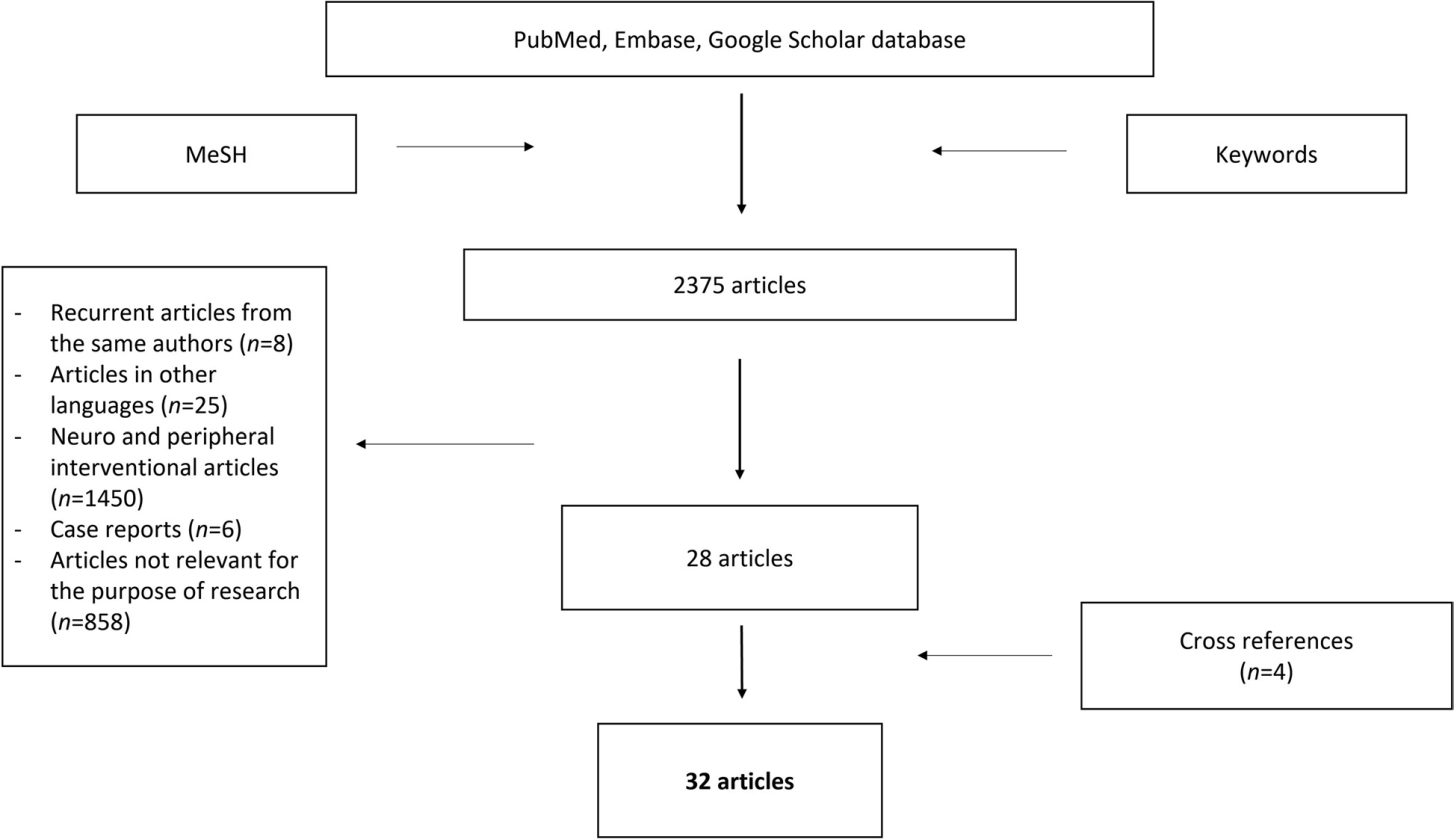
Flow-chart showing selection process of the included articles

Through the PubMed, Embase and Google Scholar database, an extensive search was performed in the fields of empiric and preventive embolization for the treatment of upper and lower gastrointestinal bleedings. We used the following medical subject headings (MeSH) and keywords: “blind embolization”, “preventive embolization”, “empiric embolization”, “empiric embolisation”, “assisted embolization”. No interval in the search period was specified. The search was performed between December 2021 and January 2022. We have included only articles in English for which it has been possible to access the entire content; patients in studies had to be male or female adults treated with empiric or blind TAE for UGIB and/or LGIB. Relevant information was drawn from original articles, reference guidelines, and previous reviews; further works have been evaluated by analysing the title, the abstract, and the bibliography of the articles found. Only studies that analysed clinical and technical success rate of blind and empiric TAE for UGIB and/or LGIB were considered for our research. Technical success was defined as the correct release of the embolizing material, with angiographic evidence of occlusion of the target arteries in the absence of any signs of bleeding at the end of the procedure, while clinical success was defined as the absence of signs of bleeding on imaging or laboratory examinations during the follow-up period. Finally, publications relevant to the purpose have been selected for this review. Research of the studies was performed by two authors (C.I. and G.D.) and disagreements on literature data were resolved by consensus discussion with other authors. Exclusion criteria were: recurrent articles from the same authors, articles written in other languages, those in which the entire content could not be accessed and articles were not consistent to the purposes of our research (for non-specific use of keywords or because they are used in reference to neuro or peripheral vascular intervention) and case reports. From the initial research applying the keywords mentioned, 2375 articles were extracted. After application of the inclusion and exclusion criteria, the total number of articles selected were reduced to 28. A further 4 articles have been added to the search based on cross-references from previous reviews or original studies. The systematic analysis of the literature was then conducted on a total of 32 articles including original articles, review articles and case series. The selection process of the included articles is summarized in Fig. 1. Patients enrolled, bleeding causes, initial diagnostics approach, embolic materials and techniques, technical success, clinical success, rebleeding, survival and complication rates were recorded using Microsoft Excel database (Microsoft Corporate, Redmond, WA, USA) and empiric or target embolization were underlined in each study. The following characteristics were extracted from each study analysed: first author, year of publication, types of study design, number of patients enrolled for blind or preventive TAE, bleeding source, timing and aim of TAE, embolic material, technical and clinical success, complication and outcome consideration in a 30-day period of surveillance. The data related to technical success, clinical success and complications were comparatively evaluated. To indicate complications, with or without clinical implications, it was decided to standardize what was declared by the authors of the individual studies using the CIRSE classification system of complications (Filippiadis et al. 2017). Whenever possible, subgroups of patients undergoing treatment with specific embolic materials were indicated. All the data analysed was included when declared by the authors of the single studies or in any case derivable from the material provided in the articles; in doubtful cases, "undeclared" was indicated. Continuous variables were presented as means and standard deviations, and categorical variables were presented as percentages. It was chosen to compare the proportions of the outcomes of the studies using Freeman-Tukey transformation to calculate the weighted summary proportion under the fixed and random effects model, as reported in the technical guide of the software used (Barker et al. 2001). An attempt at meta-analysis has been made, but the lack of homogeneity in the presentation of the results, mainly with regard to LGIB, has discouraged this aim. The study did not directly involve humans, so does not require the Institutional Review Board approval of our department.

## Results

We collected pooled data on 1019 patients from 32 separate articles selected according to the inclusion and exclusion criteria. We collected 22 studies focusing on UGIB (total 773 patients), one article focusing on LGIB (total 6 patients) and 9 studies that enrolled patients with both UGIB and LGIB (total 240 patients) (Aina et al. 2001; Arrayeh et al. 2012; Boros et al. 2021; Defreyne et al. 2001; Dempsey et al. 1990; Dixon et al. 2013; Encarnacion et al. 1992; Feld et al. 2010; Foley et al. 2010; Green et al. 2005; Han et al. 2017; Heianna et al. 2014; Holme et al. 2006; Ichiro et al. 2011; Kaminskis et al. 2017; Kaminskis et al. 2019; Katano et al. 2012; Kim et al. 2009; Kobayashi et al. 2004; Lang et al. 1992; Lau et al. 2019; Laursen et al. 2014; Lee et al. 2012; Ljungdahl et al. 2002; Mille et al. 2015; Morris et al. 1986; Muhammad et al. 2019; Padia et al. 2009; Peynircioğlu et al. 2011; Poultsides et al. 2008; Rachapalli and Nagabhushan 2018; Rösch et al. 1972; Schenker et al. 2001; Schuetz and Jauch 2001; Shi et al. 2017; Sildiroglu et al. 2014; Song et al. 2011; Spiliopoulos et al. 2018; Tandberg et al. 2012; Tipaldi et al. 2018; Toyoda et al. 1995; Warneke et al. 2004; Yap et al. 2013; Yata et al. 2013; Yu et al. 2021; Zhou et al. 2013). Five studies analysed indications and technique of preventive TAE. With regard to the typology of the experimental design, we collected 24 retrospective series (none of these specifically analysed LGIB, but eight of these were mixed UGIB and LGIB series), 3 prospective studies (one of which with mixed UGIB and LGIB series), 2 randomized trials (UGIB related series only); we also included in our analysis 3 case series (these were all blind TAE of UGIB). Main characteristics of the studies used for the systematic review were compared and summarized in the Table 1. Timing of TAE was reported in 31 studies. In 23 papers, TAE was performed after endoscopic intervention (with or without technical success), while in 5 studies TAE was performed after CT angiography. In 3 studies TAE attempts were performed directly on the basis of clinical suspicion. We analysed different types of embolic agents, which have been used in blind and/or preventive TAE procedures. Only a few articles have highlighted the numbers of patients treated with specific embolic materials; in four articles, authors do not specify the embolic material used in their series. Six articles show the exclusive use of coils. All other articles report the combined use of various embolizing agents, including microspheres, PVA particles, coils, EVHO and NBCA, in various combinations (Tipaldi et al. 2018). The source of bleeding was peptic ulcer lesions in eight studies, while in four studies, the term “lesion” was used as a generic term for various bleeding sources (e.g., angiodysplasia, solid tumors, peptic ulcers). Gastroduodenal artery was the main vessel embolized followed by left gastric artery and right gastric artery for UGIB, while superior rectal arteries were the main vessels embolized for LGIB. Vessels embolized and main embolic materials used for UGIB and LGIB were collected and summarized in Table 2. Excluding the articles for which data were not directly derivable, results on technical and/or clinical success were available for 783 patients from 27 different studies summarized in Table 3. All cases of UGIB and LGIB were compared in terms of technical success and clinical outcome. Technical success rate varied from 62% to 100% (Han et al. 2017; Heianna et al. 2014; Ichiro et al. 2011; Katano et al. 2012; Kim et al. 2009; Tandberg et al. 2012; Tipaldi et al. 2018), with a mean value of 97.7% comparing the data with Freeman-Turkey transformation for meta-analysis of the dataset (fixed affect); the clinical success rate varied from 51% to 100% (Feld et al. 2010; Heianna et al. 2014; Ichiro et al. 2011; Katano et al. 2012; Morris et al. 1986; Sildiroglu et al. 2014; Tandberg et al. 2012) with a mean value of 80%. Our analysis based on the comparison of studies found no significant differences in outcomes between UGIBs and LGIBs when comparing technical and clinical success.

**Table 1:** Characteristics of the studies used for the systematic review

**First author, year**	**Number of patients**	**Gastrointestinal bleeding location (Upper/Lower)**	**Type of Study design**
Aina et al., 2001 (2001)	29	Upper	retrospective
Arrayeh et al., 2012 (2012)	56	Upper	retrospective
Defreyne et al., 2001 (2001)	6	Upper and lower	retrospective
Dempsey et al., 1990 (1990)	39	Upper	retrospective
Dixon et al., 2013 (2013)	20	Upper	retrospective
Encarnacion et al., 1992 (1992)	29	Upper	retrospective
Heianna et al., 2014 (2014)	6	Lower	retrospective
Holme et al., 2005 (2006)	28	Upper	prospective
Ichiro et al., 2011 (2011)	36	Upper	retrospective
Kaminskis et al., 2019 (2018)	58	Upper	prospective
Katano et al., 2012 (2012)	2	Upper	retrospective
Kim et al., 2009 (2009)	75	Upper and lower	retrospective
Lang et al., 1992 (1992)	7	Upper	case series
Lau et al., 2019 (2019)	96	Upper	randomized controlled trial
Laursen et al., 2014 (2014)	49	Upper	randomized controlled trial
Lee et al., 2012 (2012)	10	Upper and lower	retrospective
Ljungdahl et al., 2002 (2002)	4	Upper	retrospective
Mille et al., 2015 (2015)	75	Upper	retrospective
Morris et al., 1986 (1986)	9	Upper	case series
Muhammad et al., 2019 (2019)	32	Upper and lower	retrospective
Padia et al., 2009 (2009)	72	Upper	retrospective
Peynircioglu et al., 2011 (2011)	42	Upper and lower	retrospective
Poultisides et al., 2008 (2008)	22	Upper	retrospective
Schenker et al., 2001 (2001)	103	Upper	retrospective
Shi et al., 2017 (2017)	5	Upper and lower	prospective
Sildiroglu et al., 2014 (2014)	18	Upper	retrospective
Song et al., 2011 (2011)	6	Upper	case series
Spiliopoulos et al., 2018 (2018)	10	Upper	retrospective
Tandberg et al., 2012 (2012)	25	Upper and lower	retrospective
Toyoda et al., 1995 (1995)	5	Upper	retrospective
Yap et al., 2013 (2013)	38	Upper and lower	retrospective
Yata et al., 2013 (2013)	7	Upper and lower	retrospective

**Table 2:** Embolic agents used in each study and vessels treated

**First author, year**	**Number of patients**	**Vessel treated**	**Embolic materials** ^a^
Aina et al., 2001 (2001)	29	GDA (21) LGA (8)	Coils, glue, PVA particles with or without gelfoam
Arrayeh et al., 2012 (2012)	56	LGA (31) RGA (5)	Gelfoam (7 patients), PVA particles (2 patients), coils (28 patients), gelfoam and PVA particles (2 patients), Coils and gelfoam (17 patients)
Defreyne et al., 2001 (2001)	6	LGA (3), GDA (2), IPDA (1)	Coils, gelfoam
Dempsey et al., 1990 (1990)	39	LGA (15), GDA (3), IPDA (1), CHA (1), RGA (1)	Coils and/or gelfoam
Dixon et al., 2013 (2013)	20	GDA (20)	Coils (17 patients), coils and gelfoam or PVA particles (3 patients)
Encarnacion et al., 1992 (1992)	29	LGA (14), GDA (11), SMA (6)	Coils, gelfoam, PVA particles
Heianna et al., 2014 (2014)	6	Vasa recta (6)	Gelfoam and/or microcoils
Holme et al., 2005 (2006)	28	GDA (28)	Coils
Ichiro et al., 2011 (2011)	36	GDA (36)	Coils
Kaminskis et al., 2019 (2019)	58	GDA and LGA (58)	Coils
Katano et al., 2012 (2012)	2	LGA (1)	Coils
Kim et al., 2009 (2009)	75	-	Undeclared
Lang et al., 1992 (1992)	13	LGA (7)	Gelfoam, Coils
Lau et al., 2019 (2019)	96	GDA (63), LGA (18), RGA (11)	Coils and/or gelfoam
Laursen et al., 2014 (2014)	49	GDA (49)	undeclared
Lee et al., 2012 (2012)	10	GDA (15), HA (1), LGA (2), RGEA (1), JA (1), IIA (1)	Coils
Ljungdahl et al., 2002 (2002)	4	GDA (4)	Coils, microcoils and/or gelfoam
Mille et al., 2015 (2015)	75	GDA (55)	Coils and/or NBCA
Morris et al., 1986 (1986)	9	LGA (9)	Gelfoam and/or microcoils
Muhammad et al., 2019 (2019)	32	GDA (24), IA (3), LGA (2) RGEA (1), MRA (1)	Coils, PVA particles and/or gelfoam (in various combination)
Padia et al., 2009 (2009)	72	GDA (64, LGA (13)	Coils
Peynircioglu et al., 2011 (2011)	42	LGA (3)	Coils, PVA particles
Poultisides et al., 2008 (2008)	22	-	Coil and microcoils, gelfoam, PVA
Schenker et al., 2001 (2001)	103	GDA and LGA (103)	Gelfoam and/or microcoils, occasionally anche polivynil alcool
Shi et al., 2017 (2017)	5	-	coils, PVA, embosphere
Sildiroglu et al., 2014 (2014)	18	LGA (2), GDA (13), GEA (2), PDA (1)	Coils (7 patients); gelfoam (3 patietns); coils and gelfoam (6 patients); coils, gelfoam and particelle (1patient); gelfoam and particelle (1 patient)
Song et al., 2011 (2011)	6	GDA (1), LGA (8)	Coils (4 patients), gelfoam (2 patients)
Spiliopoulos et al., 2018 (2018)	10	GDA and LGA (10)	undeclared
Tandberg et al., 2012 (2012)	25	-	Microcoils, PVA particles
Toyoda et al., 1995 (1995)	5	-	Coils and/or gelfoam
Yap et al., 2013 (2013)	38	-	Coils (23 patients), coils and gelfoam (7 patients), PVA (8 patients)
Yata et al., 2013 (2013)	7	-	NBCA (6 patients), coils (1 patient)

*GDA* Gastroduodenal artery, *LGA* Left gastric artery, *RGA* Right gastric artery, *IA* Ileocolic artery, *IPDA* Inferior pancreatic duodenal artery, *CHA* Common hepatic artery, *SMA* Superior mesenteric artery, *JA* Jejunal artery, *IIA* Internal iliac artery, *HA* Hemorroidal artery, *RGEA* Right gastroepiploic artery, *MRA* Middle rectal artery, *PDA* Pancreaticduodenal artery

^a^In brackets the number of patients, when specified by the Authors, for each material

**Table 3:** Clinical and technical success rate in each study analysed

**First author, year**	**Number of patients**	**Technical success rate (%)**	**Clinical success rate (%)**
Aina et al., 2001 (2001)	29	98.7%	76%
Arrayeh et al., 2012 (2012)	56	-	-
Defreyne et al., 2001 (2001)	6	100%	83%
Dempsey et al., 1990 (1990)	39	-	-
Dixon et al., 2013 (2013)	20	95%	80%
Encarnacion et al., 1992 (1992)	29	62%	82%
Heianna et al., 2014 (2014)	6	100%	100%
Holme et al., 2005 (2006)	28	100%	61%
Ichiro et al., 2011 (2011)	36	100%	83%
Kaminskis et al., 2019 (2019)	58	100%	96.6%
Katano et al., 2012 (2012)	2	100%	100%
Kim et al., 2009 (2009)	75	100%	86%
Lang et al., 1992 (1992)	7	100%	86%
Lau et al., 2019 (2019)	96	100%	94%
Laursen et al., 2014 (2014)	49	-	-
Lee et al., 2012 (2012)	10	-	-
Ljungdahl et al., 2002 (2002)	4	100%	100%
Mille et al., 2015 (2015)	75	98%	87%
Morris et al., 1986 (1986)	9	100%	67%
Muhammad et al., 2019 (2019)	32	96.9%	92%
Padia et al., 2009 (2009)	72	-	-
Peynircioglu et al., 2011 (2011)	42	100%	62.5%
Poultisides et al., 2008 (2008)	22	94%	51%
Schenker et al., 2001 (2001)	103	95%	75%
Shi et al., 2017 (2017)	5	100%	60%
Sildiroglu et al., 2014 (2014)	18	100%	67%
Song et al., 2011 (2011)	6	100%	67%
Spiliopoulos et al., 2018 (2018)	10	100%	-
Tandberg et al., 2012 (2012)	25	100%	68%
Toyoda et al., 1995 (1995)	5	100%	80%
Yap et al., 2013 (2013)	38	99%	76%
Yata et al., 2013 (Yata et al. 2013)	7	100%	96%

The total number of complications for UGIB e LGIB was 57 events out of 1019 procedures analysed; the following minor complications were found: bleeding from the access site and abscess at the access site; the following major complications have been described: coil migration, intestinal ischemia, hepatic abscess, massive hematemesis following heparin administration, gastroesophageal junction perforation, gastric artery dissection (Mille et al. 2015; Schenker et al. 2001; Spiliopoulos et al. 2018). According to the CIRSE system classification, 18 events were classified as grade 1, 14 events as grade 2, 11 events as grade 3 and 3 events as grade 4. Number, rate and type of complications reported in the individual studies used for the systematic review were collected in Table 4. The overall proportions of complications were 4.08%, considering the total number of the procedures and the cumulative complications (pooled data from UGIB and LGIB). After being separately assessed, the pooled incidence of complications in patients with UGIB and LGIB who underwent blind and/or preventive TAE procedure was approximately 1.13% in UGIB and 9.77% in LGIB.

**Table 4:** Rate and type of complications reported in the individual studies

**First author, year**	**Number of patients**	**Complications rate %**	**Complications** **(number of patients)**
Aina et al., 2001 (2001)	29	0%	-
Arrayeh et al., 2012 (2012)	56	2%	Bleeding access site (1)
Defreyne et al., 2001 (2001)	6	0%	No complication reported
Dempsey et al., 1990 (1990)	39	0%	No complication reported
Dixon et al., 2013 (2013)	20	0%	No complication reported
Encarnacion et al., 1992 (1992)	29	14%	Minor complication required no treatment
Heianna et al., 2014 (2014)	6	67%	Minor intestinal ischemia (4)
Holme et al., 2005 (2006)	28	0%	No complication reported
Ichiro et al., 2011 (2011)	36	0%	No complication reported
Kaminskis et al., 2019 (2019)	52	0%	No complication reported
Katano et al., 2012 (2012)	2	0%	No complication reported
Kim et al., 2009 (2009)	75	0%	No complication reported
Lang et al., 1992 (1992)	7	0%	No complication reported
Lau et al., 2019 (2019)	10	0%	No complication reported
Laursen et al., 2014 (2014)	49	0%	No complication reported
Lee et al., 2012 (2012)	25	28%	Duodenal necrosis (1), gastresophageal junction perforation (1), large bowel ischemia (1), massive hematemesis following heparin challenge (1), access site haematoma (3)
Ljungdahl et al., 2002 (2002)	18	0%	No complication reported
Mille et al., 2015 (2015)	75	4%	The major complication rate was 4%.
Morris et al., 1986 (1986)	9	11%	Gastric artery dissection (1)
Muhammad et al., 2019 (2019)	32	12%	Coil migration (3 patients), access site hematoma (1 patient)
Padia et al., 2009 (2009)	72	1%	3 coils misplaced and 1 groin hematoma
Peynircioglu et al., 2011 (2011)	42	0%	No complication reported
Poultisides et al., 2008 (2008)	22	0%	No complication reported
Schenker et al., 2001 (2001)	103	13%	Major complication in 3%, minor complication in 10,4%
Shi et al., 2017 (2017)	5	0%	No severe complication reported
Sildiroglu et al., 2014 (2014)	18	22%	3 coils displacement, 1 haematoma at access site
Song et al., 2011 (2011)	6	0%	No complication reported
Spiliopoulos et al., 2018 (2018)	10	5%	The rate of major complications was 4.5%
Tandberg et al., 2012 (2012)	25	0%	No complication reported
Toyoda et al., 1995 (1995)	6	0%	No complication reported
Yap et al., 2013 (2013)	38	16%	Coils migration in hepatic artery (3), splenic artery (2), hepatic infarction (1)
Yata et al., 2013 (2013)	7	86%	Postembolization gastric ulcer

## Discussion

The role of the “blind” TAE has been extensively studied for the treatment of gastrointestinal bleeding representing an effective and valid alternative to surgical treatment. In most cases, an endoscopy would be the first choice to diagnose and treat a GIB. Blind TAE refers to patients without direct signs of bleeding on angiography but with clinical or laboratory findings of bleeding or with indirect sign of bleeding on angiography, like aneurysms or pseudoaneurysms, vessel irregularity, cut-off vessel sign, alteration in regional vascularity of gastrointestinal tract (arteriovenous shunting, neovascularity, increased vascularity from dilated arterioles) or patients with positive CT findings of arterial bleeding or in those for whom endoscopic treatment has not been effective (Yu et al. 2021). TAE was introduced in 1972 by Rosch (Rösch et al. 1972) and ever since has been widely used in the management of intestinal bleeding, particularly in acute patients for whom an endoscopic treatment option has failed or was not suitable due to their critical conditions, the inability to obtain adequate bowel preparation and in patients at high risk of re-bleeding (Expert Panels on Vascular Imaging and Gastrointestinal Imaging et al. 2017; Strate and Gralnek 2016). Consolidated literature showed that in the case of UGIB the arteriography performed in the acute phase detects bleeding in about 80% of cases, it is not influenced by the lack of visualization of the blush in the intestinal lumen and bleeding of 0.3ml/min may be demonstrated; arteriography has a high sensitivity to bleeding of arterial or capillary origin, while it has low sensitivity to bleeding of venous origin and intermittent bleeding (Song et al. 2011). TAE procedure is very useful in patients with active and visible UGIB, with very high technical success and favourable clinical outcomes in about 80% of cases, a low rate of complications and less recourse to surgery (Aina et al. 2001); TAE is also the method of first choice in patients with post-surgical or post-traumatic UGIB, since these patients are not amenable to a safe endoscopic approach (Spiliopoulos et al. 2018; Zhou et al. 2013). The indication for TAE in LGIBs arises in particular for the treatment of acute ongoing bleeding and high clinical risk that do not respond adequately to systemic therapy or that could not tolerate intense purge to prepare the intestine or could not undergo colonoscopy (Strate and Gralnek 2016). It was reported that the cumulative complication rate in TAE for LGIB is relatively high (up to one third of treated patients) and intestinal ischemia occurs in up to 4% of cases (Yata et al. 2013). For these reasons, TAE for LGIB has so far found an indication in acute treatments that cannot be managed in any other way, and blind procedures have rarely been reported in the literature due to the risk of complications. Our analysis of the literature showed that most of the published series on blind or preventive TAE procedures focused on the treatment of bleeding proximal to Treitz’s ligament, as we expected given the indications of the current commonly accepted guidelines and in relation to the lower expected frequency rate of adverse events in this vascular territory compared to the lower gastrointestinal tract. Only few articles on blind/preventive embolization for LGIB have been reported, most of which are case series only, and therefore the level of evidence is rather poor (Expert Panels on Vascular Imaging and Gastrointestinal Imaging et al. 2017; Strate and Gralnek 2016). In this literature review, we evaluated the type of embolic agents used for the blind and preventive treatment of intestinal bleeding. A variety of embolic agents has been used, both singly and in various combinations. We have collected articles with the use of coils and microcoils, gelfoam, PVA, NBCA and EVOH. Coils are the preferred embolizing material by the authors, followed by gelfoam and PVA. Since the proportion of patients for whom a specific material was used was not reported in most of the articles selected, no conclusions could be drawn regarding the efficacy and complications of the individual materials. However, it should be noted that there is no clear evidence of the superiority of an embolizing material over others, and indeed there are conflicting reports on this considering different materials with the same effectiveness (Kim et al. 2009; Tipaldi et al. 2018). Embolic materials present different complications. Gelfoam is a temporary agent, burdened with a risk of rebleeding and non-target embolization; Heianna et al., in their series on blind TAE for LGIB, found that the migration of gelfoam was the main cause of several intestinal ischemia, although this complication did not require urgent surgical treatment (Heianna et al. 2014). Coils could migrate with non-target embolization if not released correctly and the treated vessel cannot be embolized if rebleeds. Glues require changing of catheter after use and experience in release, while sclerosant agents are poor visible on angiography, painful and with high risk of non-target embolization. Most of the comparable data between the various studies was related to technical success, clinical outcome and complications, as most of the authors of the consulted articles expressed them clearly in their papers. Definitions of clinical success and complications could vary in articles collected, nevertheless we were able to derive comparable data in most cases. If the data relating to UGIB and LGIB are considered cumulatively, the technical success of blind or preventive embolization (defined as the correct release of the embolizing material, with angiographic evidence of occlusion of the target arteries in the absence of any signs of bleeding at the end of the procedure) appears to be very high with a pooled average of 97.7%; pooled clinical success (which we considered as the absence of obvious bleeding or signs of bleeding on imaging or laboratory examinations during the follow-up period) was 80%. These data are substantially comparable to what has already been reported in consolidated literature regarding the interventional treatment of intestinal bleeding above Treitz’s ligament (Aina et al. 2001) and because the outcomes seem to be statistically not different between blind and preventives of UGIB and LGIB, opening up prospects for blind treatment also in this anatomic region for which there is currently no clear indication in the literature. Target vs blind TAE have been directly compared in some original studies we reviewed. Arrayeh et al. described that in the case of gastric bleeding the clinical success at thirty days was superior in patients in whom the origin of the bleeding was angiographically identified compared to patients treated blind, but there were no statistically significant differences in outcomes between blind and overt patients with duodenal bleeding (Arrayeh et al. 2012); Dafreyne et al., evaluating a case series including both UGIB and LGIB, described that no statistically significant difference there was between blind embolization treatment and cases with a known bleeding source even in patients with early rebleeding after TAE procedure (Defreyne et al. 2001); comparing TAE procedure in two groups of patients (blind embolization vs target embolization) different authors highlighting there was no statistically significant difference in 30-day mortality, reoperation or recurrence of bleeding in the two groups (Dixon et al. 2013; Ichiro et al. 2011; Kaminskis et al. 2019; Mille et al. 2015). Regarding preventive TAE, Katano et al. reported that Rockall score> 7 and Forrest class Ia/Ib were an independent prognostic factors of endoscopy failure and in these patients there was greater utility of preventive embolization (Holme et al. 2006; Katano et al. 2012); in a post hoc analysis of a case series that compared patients treated for preventive TAE and untreated patients after endoscopy for gastro-duodenal ulcer, Lau et al. stated that there is an advantage in embolizing preventively in the presence of ulcers > 15 mm, and that the re-bleeding rate is slightly higher (but not statistically significant) in untreated cases compared to those treated with TAE (Lau et al. 2019); furthermore preventive TAE in patients with gastro-duodenal ulcer is associated with a shorter duration of hospitalization and the re-bleeding rate (Boros et al. 2021; Kaminskis et al. 2017; Laursen et al. 2014). Sildiroglu et al. defined that no statistically significant difference in terms of clinical success or complications between patients postponed to blind / preventive TAE and therapeutic TAE (Sildiroglu et al. 2014). Tandberg et al. in a retrospective analysis that included patients with UGIB or LGIB stated that patients with tumour-induced bleeding have greater success from blind TAE, with no significant difference between hyper and hypo vascularized tumours, and concluded that TAE, even if without evidence of contrast media extravasation, could be a possible palliative therapy in neoplastic patients presenting with tumor bleeding (Dempsey et al. 1990; Lee et al. 2012; Shi et al. 2017; Tandberg et al. 2012). Yap et al., focused their attention on patients with LGIB, demonstrating that the rebleeding rate was similar in empirical embolization compared to active extravasation embolization, but also founding higher mortality rate in the empirically treated group, likely related to surgical management of the complications (Foley et al. 2010; Schuetz and Jauch 2001; Yap et al. 2013). The analysis of complication data takes into account both major complications (including, as mentioned, intestinal ischemia, but also hepatic or splenic ischemia or other sites for migration of coils or other embolic material and vascular dissection), and also of minor complications (including hematoma of the access site, non-occlusive gastric artery dissection which no require further treatment and others). The pooled complication rate (UGIB and LGIB) was 4.20% and this seems to be in line with what has already been reported in the literature for the treatment of upper tract bleeding (Aina et al. 2001; Lang et al. 1992; Ljungdahl et al. 2002; Muhammad et al. 2019; Padia et al. 2009). The analysis of complication rates in LGIBs subjected to blind TAE procedures demonstrates a cumulative incidence of adverse effect of 9.77% with a difference significant compared to complications for UGIB treatments. The complications found in these patients are various, and also include intestinal ischemia, which can be potentially fatal and often requires surgical treatment. This is also due to the poor representation of the arterial collaterals in the lower gastrointestinal tract that increase the risk of ischemia after embolization compared to UGIB (Green et al. 2005).

## Limits

Our systematic review present two main limitations. Analysis of the data collected was mainly based on retrospective studies, most of which referred to UGIB; only a few articles were case series on LGIB, while some data were extrapolated from "mixed" series (which treating both UGIB and LGIB blind procedures). This is represents a limit in terms of the scientific evidence for the biases common to all retrospective studies and for the clinical and statistical inhomogeneity that can emerge from a pooled analysis of data; however, this limit was expected, since it is complex to plan clinical trials or prospective studies concerning potentially life-threatening conditions. In fact, there are no systematic reviews in the literature on both types of bleeding. To the best of our knowledge, only three randomized trials with direct comparison of outcomes between blind and non-blind embolization have been reported for UGIB and none for LGIB: this has discouraged a meta-analysis on this point, being able to express only on cumulative data relating to technical and clinical success and complications. Furthermore, technical and clinical success rate were extrapolated from the analysis of retrospective, prospective and case series studies; the latter, however, have low level of scientific evidence. This represents the second, and probably the main, limitation of our study.

## Conclusions

TAE is an effective procedure in the treatment of UGIB patients with bleeding source not detected on endoscopy or who cannot undergo endoscopy, or in which angiography does not demonstrate direct sign of ongoing bleeding. The clinical success and complications of this approach is substantially comparable to patients with positive angiography; preventive TAE could find application in selected populations due to the high risk of rebleeding, also in consideration of the generally limited complications in empirical embolization of the upper gastrointestinal tract. The attitude in the treatment of LGIBs must be more prudent: although also in this case the guidelines recommend the use of angiography in case of bleeding that cannot be managed endoscopically, a treatment as selective as possible is recommended, in relation to poor vascular anastomoses and the high risk of intestinal ischemia; in case of failure to visualize the source of the bleeding, there are no clear indications in favour of blind or preventive embolization in this district and therefore the choice must be based on the clinical-radiological findings and the effective possibility of quickly treating any complications in the environment surgically. Blind and preventive procedures cumulatively present a relatively low risk of complications, compared to a relatively high technical and clinical success. The results relating to what is reported on UGIB are substantially overlapping with what has already been reported in other previous reviews, and do not seem to require further confirmation. On the other hand, few data are available on the blind and preventive treatment of LGIBs, and the inhomogeneity of the experimental designs proposed by the authors does not allow us to propose a meta-analysis. There is therefore a lack of evidence on LGIBs, and in-depth studies and trials for this specific anatomical district are necessary.

## Data Availability

Not applicable.
